# The Paradoxical Impacts of the Minimum Wage Implementation on Ready-made Garment (RMG) Workers: A Qualitative Study

**DOI:** 10.1007/s41027-022-00375-9

**Published:** 2022-08-01

**Authors:** Humayun Kabir, Myfanwy Maple, Md. Shahidul Islam, Kim Usher

**Affiliations:** 1 School of Health, Faculty of Medicine and Health, University of New England, Armidale, NSW 2351 Australia; 2 University of Dhaka, Dhaka, 1000 Bangladesh

**Keywords:** Minimum wages, Ready-made garment workers, Profit maximization, Health and well-being, Bangladesh, Qualitative study

## Abstract

There is no regular mandated increase in minimum wages for workers employed in the Bangladesh ready-made garment (RMG) industry. Workers in the past have relied on optional bonuses added to their monthly incomes to supplement their wages. However, a new minimum wage implemented in January 2019 in the Bangladesh RMG sector increased wages for many workers who are known to work under poor and exploitative working conditions. Qualitative in-depth interviews were conducted with fifteen currently employed RMG workers (female: 13, male: 2), which led to data saturation. The participants were purposively recruited from both export processing zone (EPZ) and non-EPZ factories located in Dhaka and Chattogram, the two largest cities of Bangladesh where the majority of RMG factories are situated. Transcribed interviews were analysed thematically. The findings revealed that working hours, production targets, work pressure, and workplace abuse have an impact on workers’ health and well-being. In line with the Marxist notion of the “accumulation of capital”, we argue that due to the profit maximization mindset of RMG owners and international brands, workers have not received the potential benefit of the newly implemented minimum wage as their conditions have been changed in other ways to offset the increase in salary. The article contributes to understanding how factory owners’ profit maximization mindset dispossessed workers from receiving the real benefits of the newly implemented minimum wage and forced them to continue working within exploitative working environments. The study shows that the impact of minimum wages on poverty reduction is unlikely and outline the need for RMG labour market reform.

## Introduction

The rapid expansion of the Bangladeshi ready-made garment (RMG) sector has opened huge work opportunities for more than 4 million workers (Syed 2020; Leitheiser et al. 2020), mostly female, often from rural and/or poor socio-economic backgrounds, with little or no education, semi-skilled/unskilled, and with limited/no access to other professions (Alamgir and Banerjee 2019; Richards et al. 2016; Stanwick and Stanwick 2015; Azad 2014). This sector is now considered the biggest export earner, with a value of over $27.9 billion of exports in the 2019–20 financial year in Bangladesh (Bangladesh Garment Manufacturers and Exporters Association (BGMEA) 2021). The Bangladesh RMG sector has not only economically benefitted the country, but has also provided many benefits the factory owners since the 1980s. Bangladeshi RMG factory owners manage to supply clothing products for the international clothing brands at a lower price compared to other countries such as China, India, Vietnam, Cambodia, Pakistan, and Thailand (Moazzem and Arfanuzzaman 2018; Cowgill and Huynh 2016; Kurpad 2014). This is mainly due to paying low wages to the RMG workers in Bangladesh (Ashraf and Prentice 2019; Kabir et al. 2019a, b). To ensure low costs for the international brands, the factory owners tend to cut expenses where possible, especially related to expenditures required to ensure a safe and healthy workplace and reasonable wages for the RMG workers.

Before the implementation of the recent minimum wages for Bangladeshi RMG workers in 2019, the government had only increased the minimum wages for the workers three times; in 1994, 2006, and 2010 since the commencement of the sector in the 1980s (Ahmed and Nathan 2016). The total amount of wage increases from one to another minimum wage implementation period (see Table 1) raises important questions about whether the new minimum wage could reduce the ongoing poverty of RMG workers, an ongoing debate in developing countries where the RMG industry has prospered (Rani 2017; Aderemi 2017).

**Table 1:** Increase in minimum wages since commencement of Bangladesh RMG sector [Quoted from Ahmed and Nathan (2016); *The Daily Star*, 14 January 2019]

Year (1)	Minimum Wage (2)	Increase in Wages (3)
1994	BDT 930 (USD 11)	–
2006	BDT 1,662.5 (USD 20)	BDT 732.5 (USD 9)
2010	BDT 3,000 (USD 36)	BDT 1337.5 (USD 16)
2019	BDT 4,100 (USD 49)	BDT 1100 (USD 13)

Note: Column 3 is the result of the authors’ own calculations, and the conversation rate from BDT to USD may differ. The minimum wage reported here is applicable for the grade 7 workers (e.g. helpers/newly employed workers)

RMG workers’ minimum wages are not upgraded regularly in Bangladesh and workers have to be satisfied with a variety of bonuses [i.e. *HAJIRA* (presence) bonus/target bonus-workers receive these bonuses if they do not take any leave in a particular month and able to fulfil hourly/monthly production targets] they receive to supplement their monthly salary (Fair Wear Foundation 2012). To receive these bonuses, workers continue working to fulfil production targets, even if they are physically ill (Kabir et al. 2019a). This is indeed an exploitation/violation of workers’ rights in taking sick leave when needed. Factory owners usually introduce these bonuses to discourage workers from taking sick leave. As a result, the factory owners have been accused of being more interested in the intensification of their profits from the RMG business rather than investing money to ensure a safe workplace and quality of life for their workers (Anner 2020; Muhammad 2015; Brown 2015).

Empirical research focusing on the impacts of minimum wages on Bangladeshi RMG workers remains sparse. However, there has been an ongoing debate regarding the adequacy of the RMG workers’ wages in Bangladesh since the inception of this sector in the 1980s (Khan et al. 2020; Yasmin 2014; Khosla 2009; Salway et al. 2005; Kabeer 2004; Kabeer and Mahmud 2004; Mahmud and Kabeer 2003; Paul-Majumder and Begum 2000). Previous studies indicated that workers experience personal difficulties due to the low wages paid in the sector, including: (a) keeping children away (that is, living with extended family in rural areas to reduce costs in urban settings), (b) taking less food/nutrition than actually needed for their physical fitness (due to less buying capacity), (c) worry/feeling insecure about the future (and not able to save money from their income), and (d) living in unhealthy places/slums or semi-slum areas (Kabir et al. 2019a, b; Ahmed and Nathan 2016; Absar 2001). The main cause of these issues lies in the high profit-taking and/or profit maximization mindset of the factory owners as well as the international brands/buyers. Due to maximizing their profits, what Marx (1952) stated as the process of “accumulation of capital”, workers’ physical labour is being exploited, which is evident from their low wages compared to other neighbouring countries. Even though the minimum wage has increased, the workers have received little benefit in real terms (Khanna 2011).

Demonstrations by the RMG workers to lobby for increased wages in Bangladesh are quite common. After a recent demonstration, the government of Bangladesh implemented the new minimum wage in early 2019 (*Textile Today*, 14 January 2019; *The Daily Star*, 14 January 2019). It is timely to investigate the newly implemented minimum wages to determine if it has met the needs of the RMG workers and helped to improve their quality of life. The study aimed therefore to explore the impact of the newly implemented minimum wages policy on RMG workers. Based on the participants’ stories, this article proposes a pressure chain that exists from top to bottom in the clothing production cycle in the Bangladesh RMG sector, which is destined to accelerate RMG production by exploiting workers. Different working groups, national and international policymakers, non-governmental organizations (NGOs)/international non-governmental organizations (INGOs), labour/trade unions, and garment factory owners’ associations may use the findings of this study to take proper steps to reduce the negative impacts of the future wage structure on workers. Additionally, the findings unveil the existence of exploitation in the Bangladesh RMG sector, which would help to encourage further investigations in the RMG sector located in South and Southeast Asian regions, because workers of these regions experience similar issues (Kabir et al. 2019a, b).

## Methods

Adopting a qualitative approach, face-to-face individual in-depth interviews were conducted among the male and female RMG workers currently employed in both the export processing zone (EPZ) and non-EPZ factories located in two large cities in Bangladesh; Dhaka and Chattogram (where the majority of the RMG factories are situated). It is worth noting that EPZ factories (mainly owned by the foreigners) in Bangladesh operate with special privileges and follow strict rules, where the government have minimal interference, thus they are expected to have better working conditions (Naved et al. 2018; Ahmed et al. 2014; Khanna 2011), compared to the locally owned non-EPZ RMG factories. Workers from both EPZ and non-EPZ factories were recruited in this study to explore the impact of the newly implemented minimum wage policy. This study was conducted and reported in accordance with the consolidated criteria for reporting qualitative studies (COREQ) (Tong et al. 2007).

Eighteen workers were invited to participate in the study, of whom fifteen consented to participate (a response rate of 83%). Three participants declined to participate because of their other engagements during the scheduled interview time. Therefore, this study was carried out through in-depth interviews with fifteen workers (female: 13, male: 2). The interviews were conducted in January 2020. One-off in-depth interviews lasted approximately 45–65 min. The in-depth interviews inquired about: (i) background information of the participants, (ii) benefits received from the newly implemented minimum wages, and (iii) impacts of the newly implemented minimum wages.

All authors (HK, MM, KU, and MSI) designed the interview schedules. Interview schedules were piloted with two workers and one trade union leader, who were not included in the final interviews. No adjustment of the interview schedule was required following the pilot study. Workers were recruited from different factories to cover possible dissimilarities regarding the topic of interest. The first author organized/conducted the in-depth interviews after assuring confidentiality to the participants. He read the consent form to the participants and upon agreement, each of them signed the informed consent form. For rapport-building purposes, the interviewer started the interview with some general questions, including questions about their life before and after joining the RMG sector, their source of entertainment, family, and working life.

Interviews were conducted once in a quiet room in the nearby union offices in Dhaka and Chattogram, in the presence of the interviewer, trained research assistant (preferably from the local place), and the contact person (RMG union leader). The research assistant helped to take notes and to overcome the language barrier during the interview period. It is worth noting that workers from Chattogram speak a slightly different language compared to the mainstream Bengali language. Additionally, the presence of the union leader increased workers’ confidence to share their stories in a safe environment. Participants consented to the presence of the research assistant. The entrance into the interview room was strictly controlled after starting the interviews to ensure the interview space remained uninterrupted and confidential. The researchers had no prior acquaintance with the participants, to avoid any possible influence. The participants were recruited by following purposive and snowball sampling techniques, commonly used in previous studies on the Bangladesh RMG sector (Kabir et al. 2021a, b; Kabir et al. 2019a, 2018; Naved et al. 2018; Fitch et al. 2017, 2015; Steinisch et al. 2013). The first author had previous work experience conducting research in Bangladesh RMG settings and maintained regular communication with the on-site contact persons (Kabir 2022; Kabir et al. 2022; Kabir et al. 2021a, b; Kabir et al. 2019a; Kabir et al. 2018), who managed the required workers for the interview purposes. Specific guidelines and unstructured questionnaires were followed to conduct in-depth interviews with both male and female workers, aged 18 and above, and having a minimum 1 year work experience in the RMG sector. The fifteen in-depth interviews led to data saturation; therefore, no further participants were recruited.

An audio recorder was used for recording in-depth interviews, and a diary was also carried with the first author for note-taking purposes. At the end of each interview, the interviewer checked for the main issues/shared meaning of the interviews and discussions as it was impossible for the interviewer to share the transcribed documents with the participants due to their low literacy level and logistical issues. The first author transcribed audio recordings verbatim into Bangla (the native language of both interviewees and the interviewer) and translated them into English. Several steps were taken to ensure the reliability of the data, including the first author, checking and verifying the scripts (Bangla and English) against the audio recordings and field notes; then, a PhD student, who has expertise in both Bangla and English language, independently attested to the correctness of the transcripts.

Thematic analysis was performed to analyse the responses derived from the participants. Specific themes (which can be words, sentences, phrases, paragraphs, or even entire documents) were used to interpret the data. Using the interpretative lens, the theme identification process was completed in three rounds (1) reading the transcripts independently to be acquainted with the content; (2) identifying themes and subthemes (transcripts were coded and compared by HK and MM); and (3) applying the theme categories after refining is completed in keeping with the proposed thematic techniques (Blackstone 2012; Braun and Clarke 2006; Ryan and Bernard 2003). The process also includes identifying the meaning of the unique units and then grouping them under larger themes (Kabir et al. 2021a, b). Final reviews of the analyses, discussion on the similar and dissimilar themes, and then final decision about picking the themes were undertaken by HK and MM.

Ethical approval was sought from the Human Research Ethics Committee of the University of New England, Australia (approval number: HE19-120; approved on: 20 June 2019) prior to the study commencement.

## Results

The participants were predominantly from rural backgrounds (i.e. only participants 02 and 07 originated from the urban area), aged between 18 and 38 years at the time of the interview, and their literacy level was predominantly low (see Table 2). Five knew how to sign/put their signatures while seven out of 15 participants’ literacy level was equal to primary school level (class 1–5). In addition, only three participants (P01, P07, and P12) attended high school (class 6–10). Most of the participants (n = 10) were sewing operators and their work experiences varied from 2–14 years. Overall, EPZ and Dhaka-based factory participants were found to be in a better position in terms of the amount of wage received, date of receiving the wage, and satisfaction with the amount increased in the new gross wages compared to non-EPZ as well as the Chattogram-based participants.

**Table 2:** Participants’ socio-demographic characteristics and the outcomes of the newly implemented wage structure

Participant Code/ Sex/ Age	Marital Status/ Place of Origin	Literacy Level	Designation	Working Years	Type of Factory/ Location of Factory	Gross Salary (before new wage)	Gross Salary (after new wage)	Amount of Wage Increased/ Wage Received Date	Satisfaction with Increased Amount	Positive Outcomes of the Newly Implemented Minimum Wages	Negative Outcomes of the Newly Implemented Minimum Wages
P01/F/30	Married/ Rural	High School	Finishing	9 years	Non-EPZ/ Dhaka	BDT 8500	BDT 10,500	BDT 2000/ By the 13^th^ of every month	No	Increased food buying capacity	Management misbehaviour Cut off snack break time Added work pressure
P02/F/32	Married/ Urban	Primary school	Sewing operator	10 years	EPZ/ Dhaka	BDT 12,000	BDT 15,500	BDT 3500/ By the 7^th^ of every month	Yes	Satisfaction regarding the amount of salary increase	Extra workload Management misbehaviour Want to quit the job
P03/F/38	Married/ Rural	Can sign only	Washing	14 years	EPZ/ Chattogram	BDT 11,000	BDT 15,000	BDT 4000/ By the 3^rd^ of every month	Yes	A bit of extra accommodation allowance added Can save little money for future	Production target has been increased Verbal abuse
P04/F/36	Married/Rural	Can sign only	Sewing operator	10 years	EPZ/ Chattogram	BDT 10,000	BDT 13,000	BDT 3000/ By the 12^th^ of every month	Yes	Eat better than before The presence bonus increased	Management misbehaviour Production target has been increased
P05/F/21	Married/ Rural	Primary school	Sewing operator	4 years	Non-EPZ/ Chattogram	BDT 7000	BDT 9500	BDT 2500/ By the 7^th^ of every month	No	Can save little money for future Eat better than before	Extra workload Want to quit the job Stress
P06/F/29	Married/ Rural	Can sign only	Sewing operator	5 years	Non-EPZ/ Chattogram	BDT 8500	BDT 10,500	BDT 2000/ By the 9^th^ of every month	No	Nothing to mention	Verbal abuse -Sick leave decreased
P07/F/18	Un-married/ Urban	High School	Sewing operator	4 years	Non-EPZ/ Chattogram	BDT 7500	BDT 9000	BDT 1500/ By the 7^th^ of every month	No	Nothing to mention	Management misbehaviour Production target has been increased Stress
P08/F/27	Married/ Rural	Primary school	Sample	11 years	EPZ/ Dhaka	BDT 14,400	BDT 16,000	BDT 1600/ By the 7^th^ of every month	Yes	Nothing to mention	Extra workload Physical and mental stress
P09/F/25	Married/ Rural	Primary school	Sewing operator	6 years	EPZ/ Chattogram	BDT 8000	BDT 12,000	BDT 4000/ By the 7^th^ of every month	Yes	Can save little money for future Increased food buying capacity	Hard to get sick leave Extended working hour
P10/M/24	Married/ Rural	Can sign only	Cutting	8 years	EPZ/ Dhaka	BDT 11,500	BDT 13,000	BDT 1500/ By the 7^th^ of every month	No	Added medical facilities	Mental pressure Unfriendly workplace environment More turmoil added to the new wage structure
P11/F/31	Married/ Rural	Can sign only	Sewing operator	7 years	Non- EPZ/ Dhaka	BDT 7500	BDT 10,000	BDT 2500/ By the 13^th^ of every month	No	Nothing to mention	Management misbehaviour Relationship between workers and the management getting worse Continuous pressure
P12/F/22	Un-married Rural	High School	Sewing operator	3 years	Non- EPZ/ Dhaka	BDT 7000	BDT 9500	BDT 2500/ By the 10^th^ of every month	No	Nothing to mention	Extra workload has been imposed Verbal/physical abuses
P13/F/19	Un-married/ Rural	Primary school	Helper	2 years	Non-EPZ/ Chattogram	BDT 5000	BDT 7000	BDT 2000/ By the 17^th^ of every month	No	Can save little money for marriage	Added work pressure Verbal abuse Fear of losing job
P14/M/23	Married/ Rural	Primary school	Sewing operator	5 years	Non-EPZ/ Dhaka	BDT 8500	BDT 10,000	BDT 1500/ By the 15^th^ of every month	No	Nothing to mention	Production target has been increased Stress Management misbehaviour
P15/F/20	Un-married/ Rural	Primary school	Sewing operator	3 years	Non-EPZ/Chattogram	BDT 8000	BDT 9500	BDT 1500/ By the 9^th^ of every month	No	Nothing to mention	Sick leave decreased Production target has been increased Verbal/physical abuses

Quoting relevant statements of the participants, the following discussions broadly focus on both positive and negative outcomes of the newly implemented minimum wages. The positive and negative outcomes are grouped in Table 3.

**Table 3:** Summary of positive and negative outcomes of the newly implemented minimum wages

Positive outcomes	Partial satisfaction with the amount increased
	Food buying capacity
	A little extra money for savings
Negative outcomes	Reducing snack/prayer breaks and sick leave
	Added workload, work pressure, and extended working hours
	New production target
	Abuse by management
	Additional mental stress
	Downward relationship between workers and factory management, and the new form of the “pressure chain”

### Positive Outcomes

This study explored some notable positive outcomes of the newly implemented minimum wages, mostly related to workers’ satisfaction with the increased salary, which accelerated their food buying capacity and enabled some of them to save money for the future.

#### Partial satisfaction with the amount increased

Five of the 15 participants were more or less satisfied with the amount of money added to their previous gross salary. Examples from two participants in relation to their satisfaction regarding the amount increased after the implementation of the new minimum wages in the following ways:

*BDT 3500 has been added to my salary after the implementation of the new pay structure and I am happy with the amount increased. But I do not know how long my happiness will last... (P02, F).*

*I expected more but was still pleased with the new salary amount. It is okay for now… (P08, F).*

#### Food buying capacity

Workers’ buying capacity for daily necessities (such as fish, meat, egg, vegetables, milk, and fruit) increased after receiving the newer wage payment. Some of them reported that they could eat a variety of vegetables, fish, meat, and fruit as their salary had increased. Participant 04 explained:

*Now, I can eat better than before… I try to include meat or fish items in our meals (P04, F).*

Together with the fish and meat items, participant 09 stated that she could often buy fruit items because of having some extra money after buying other basic foods.

*Eating fruit was not possible for me before. I do sometimes buy a variety of seasonal fruits nowadays (P09, F).*

#### A little extra money for savings

Along with adding a variety of food items, some of the participants mentioned that they were trying to save some money for their future, with the aim to start an independent business (such as a small grocery shop/tea stall) after retiring from RMG work. One participant stated that she and her husband work in the same factory. After receiving the new payment, they started to save some money for the future.

*I can now save some money for the future. I and my husband wish to set up a small grocery shop in the village with that savings… (P03, F).*

However, a participant clearly mentioned that the new wage amount was not enough to reduce their poverty in the long run.

*The new wage amount is okay for the time being, but it is not enough to reduce our poverty (P06, F).*

While the above participants showed their positive attitudes with caution towards the newly implemented minimum wages, especially regarding the amount of money increased, upgraded buying capacity, and saving capacity, participant 05 was found to be anxious about how long the workers will be able to get the current benefit as the price of daily necessities usually increases very quickly.

*I am now able to buy a variety of foods, but I am anxious about the future. The price of daily necessities is increasing day by day. I do not know how long the prices will remain under my buying capacity. The yearly increment and bonuses are not enough to adjust to the price hike (P05, F).*

While the pressure on the RMG factory owners to increase wages resulted in tangible positive outcomes for workers. However, quickly those servicing the needs of the workers—food stalls, tea houses, and the like—also increased their costs, leading to workers’ anxiety about the future.

### Negative Outcomes

Paradoxically, the newly implemented minimum wages also brought adverse conditions for the RMG workers, as narrated by the study participants. These are related to a reduction in workers’ snack/prayer breaks and sick leave, added workload and work pressure, the introduction of new production targets, extended working hours, abuse by the factory management, additional mental stress, the downward relationship between workers and factory management, and the new form of the “pressure chain”. Each of these is explored below.

#### Reducing snack/prayer breaks and sick leave

Factory management has started to reduce snacks/prayer breaks after the implementation of the new payment scheme, as one participant narrated below:

*We used to get 15 minutes for the morning snacks and another 15 minutes for afternoon snacks. The factory management has cancelled the morning, evening snacks, as well as prayer breaks after the implementation of the new minimum wage (P01, F).*

In addition to reducing snack/prayer breaks, factory management was found unwilling to approve sick leave, as explained by this participant:

*We used to get 7/8 days for sick leave in a year before the implementation of the new pay scale. The factory management does not want to give us sick leave now and behaves very rudely if anyone seeks sick leave (P06, F).*

If any worker remained absent (taking permission from the management) due to illness, money from their salary had been deducted, as explained:

*I sought sick leave and the management approved for 2 days. But, at the end of the month when I received the pay slip, I found that they deducted money (for those 2 days of sick leave) from my salary. It did not happen before (P15, F).*

#### Added workload, work pressure, and extended working hours

The factory management did not only cut or reduce snack/prayer breaks and sick leave, but they also imposed more workload and work pressure.

*Work pressure has been increased. Now, the tasks of two workers have to be done by one worker. It is indeed a double workload for us now (P13, F).*

This extra pressure has also impacted workers’ physical ability to stay at work and reduced their available sleep hours and willingness to continue RMG work.

*Due to excessive work pressure added, I now get fewer sleep hours than before. Most of the time, I feel very exhausted and restless. I do not know how long I will be able to continue this job (P05, F).*

Some of the participants mentioned that they had to work for additional hours after the implementation of the new minimum wages. Since RMG work is mainly physical labour-intensive, the extended working hours are making workers’ physical health more vulnerable than before, which is evident from the statement of one participant:

*I have to work 1 hour extra every day as the factory owners are paying us more… After finishing work, I reach home at 9 pm. Then, I start to cook for the next day. I usually go to bed at 11.30 pm and wake up around 5 am to reach the factory on time at 8 am. I, very often, feel unwell and my body just cannot tolerate this hard work anymore (P09, F).*

#### New production target

Usually, RMG workers are given hourly/daily production targets to fulfil. After the implementation of the new minimum wages, they have been given a new, higher daily production target, which was considered quite impossible by the participants:

*The management of my factory has set up extended production targets. They say, “we are paying more so you need to work more” (P07, F).*

The failure in fulfilling the production target brought more suffering for them in many ways, as participants described:

*Achieving the daily production target is quite impossible for us. Thus, we are being scolded and beaten regularly (P14, M).*

#### Abuse by management

The participants mentioned that using abusive language is a common practice at their workplace, which had become more extreme coinciding with the new minimum wages. The extreme usage of vulgar words, attacking behaviours, and verbal/physical abuse made the work experience traumatic, as explained below,

*Nowadays, the supervisor misbehaves and uses abusive language with us more frequently if he finds any fault in our work (P15, F).*

Due to the changing context, the unacceptable behaviour of the factory management at the workplace reached its highest peak ever causing the following reaction from one participant:

*I do not want the new wages in the future. If the government could control the price of daily necessities, we even do not need new wages. This new payment brings more turmoil for us. I feel tired of doing a huge amount of work now. We are kept under huge mental pressure to complete the maximum amount of tasks within a minimum amount of time (P10, M).*

#### Additional mental stress

Few of the participants shared their grievances about how the upgrade in their salary had brought added challenges and mental stress at the workplace. They started to feel the stress of losing their job without notice. Losing a job in a factory is stigmatized as it is viewed as the particular workers’ incapability/failure of fulfilling production targets. Thus, other factory owners will not employ them, which is a new tension for the workers as explained below:

*The factory management continuously threatened to kick me out if the daily production target is not fulfilled. If I am kicked out of this factory, no other factory management will employ me. (P07, F).*

One participant narrated how inhumanly he feels he has been treated at his workplace:

*From the day of receiving the new payment, the factory management started to push us to work more and more. Sometimes, we are treated like animals or machines. They keep us under mental pressure (P10, M).*

#### Downward relationship between workers and factory management, and the new form of the “pressure chain”

Participants mentioned that the relationship between the workers and the factory management has become worse than before. Their relationship is maintained through a chain, one the participants described as a “pressure chain” targeted to maximize production target outputs. Each link of the chain is pressurized by the other, by the higher authority or immediate boss. This “pressure chain” is a cruel way in which to achieve maximum production targets, as narrated by participants 01, 12, and 11:

*The supervisor of my factory has become cruel to getting work done before the deadlines. I understand that there is someone who puts pressure on them too. The pressure sustains through a cruel and inhuman chain in my workplace (P01, F).*

*The relationship between factory management and workers has deteriorated after the implementation of the new minimum wages. They are ready to do anything (bad) with workers to increase production (P12, F).*

*Now, the work pressure is so extreme that the management sometimes forgets that we are not the sewing machines (that we are made of flesh and blood) (P11, F).*

According to the participants, this “pressure chain” commenced even before the execution of the new minimum wage agreement. However, after the implementation of the new minimum wage agreement, participants were treated more inhumanly (sometimes as machines) through the command of this “pressure chain”.

## Discussion

The present study describes how the newly implemented minimum wage impacted RMG workers in Bangladesh. In essence, the increase in salary has led to the implementation of notable changes in RMG workplace settings which needed to be interrogated. No doubt, the new minimum wage has added some financial benefits for the workers in the short term. For example, some of the participants were satisfied with the amount added to their previous wages which made them able to save some money for the future and buy more food items. On the other hand, reducing existing facilities (e.g. no break for snacks/prayer, deducting salary for sick leave), setting up of new production targets, and practices of abuse in case of failure to fulfil unachievable targets have led to a deterioration in the relationship between the factory management and workers. The turmoil added along with the new minimum wages were so dreadful that the participants reported feeling depressed, stressed, and doubtful about the benefits of the new wages. Overall, the study revealed that the impacts of the new minimum wage on the participants were twofold—health (physical/psychological) and overall well-being.

The study explored that instead of reducing RMG workers’ ongoing turmoil (such as excessive workload, long working hours, physical and verbal abuses, anxiety, depression, health and safety issues, and other personal restrictions and withholding of pay in the workplace settings) (Khan et al. 2020; Alamgir and Banerjee 2019; Parvin et al. 2018), the new minimum wage added further workload/work pressure, extreme abuse, a revised production target, and mental stress in the workplace settings. Along with fewer sleeping hours (due to excessive work pressure, extended working hours, and daily productions target), the participants were stressed, scared of being scolded, beaten, and fearful of losing their jobs if they failed to fulfil the production target; a definite impact on the workers’ psychological health status similarly to what has been revealed in previous studies (Gnanaselvam and Joseph 2018; Sinkovics et al. 2016; Steinisch et al. 2013). The study findings are consistent with a prior study conducted in 2011, immediately after the implementation of the previous minimum wage agreement in the Bangladesh RMG sector, where the overwhelming majority of the respondents (92%) said they had targets to fulfil, their (82%) production targets had been increased, and that they feared losing their job in case of the failure to fulfil targets (Fair Wear Foundation 2012).

Overall, the majority of the study participants were dissatisfied with the increased amount of wage and indicated that the new minimum wage actually made the workplace environment hostile and unpleasant for them. The study identified that the implementation of the minimum wages was not enough to reduce the ongoing poverty of most workers. Thus, receiving financial benefits from the minimum wage increase remained questionable due to the factors such as price hikes of daily necessities, added physical and mental suffering, and so on. The same reactions had been noted after the implementation of the previous minimum wage in 2010 (Khanna 2011). Thus, the study argues that the negative outcomes outweighed the positive outcomes of the newly implemented minimum wage in the Bangladesh RMG sector. This study proposes that RMG workers’ bargaining capacity needs to be increased through their active participation in trade union activities with the aim to establish labour rights (and to abolish additional conditions imposed on them along with the future new minimum wages at the workplace).

It is notable that the participants recruited from the EPZ factories shared a better experience regarding the impact of the new minimum wages, i.e. in the Bangladesh RMG sector, wages differ depending on whether the workers are employed in EPZ/non-EPZ factories. Whereas, in the Canadian labour market, wages may be different on the basis of the language that the worker speaks. For example, English-speaking workers receive higher payoffs compared to those who can speak only French (Bousmah et al. 2021). Our finding is consistent with the previous construction regarding EPZ-based factories—having better working conditions, less workplace violence, and better wages compared to those of non-EPZ workers (Naved et al. 2018; Ahmed et al. 2014; Murayama and Yokota 2009). This is consistent with claims that because the majority of those factories are owned by the foreigner, regulations are stricter than in the non-EPZ factories (Khanna 2011).

**Fig. 1:**
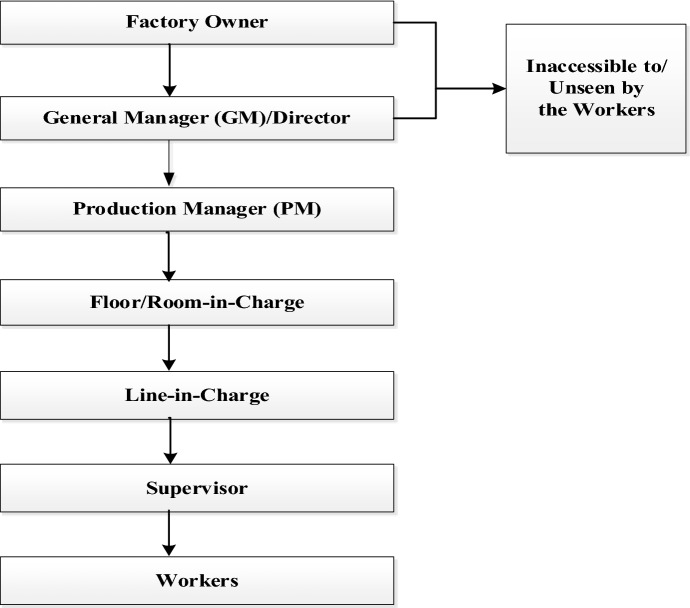
The existence of “pressure chain” in the Bangladesh readymade garment (RMG) sector

The study explored an authoritarian “chain” through which the RMG sector runs, which has become cruel and inhumane, especially after the implementation of the wage scale in 2019. The “pressure chain” system that currently exists in the RMG sector is targeted to increase production (see Fig. 1). The top management of the chain keeps pushing the immediate next management to increase the production level. The workers belong to the lowest link in the chain, and hence, they receive the greatest amount of pressure. All the upper listed personnel put pressure on the supervisor to increase production, and the supervisors turn their aggression on the workers through the use of abusive language (Akhter et al. 2019), which is indeed a human rights violation (Human Rights Watch 2015).

An improvement commensurate with the increase in wages reflected in working conditions requires monetary investments (Ahmed and Nathan 2016). However, it appears, the RMG factory owners are not interested. Rather, they prefer to maximize profit, be that cutting money from the salary, reducing break times, increasing output targets, and even removing sick leave provisions. According to Marx (1952), the capitalists/industrialists exploit workers’ labour to accumulate as much capital as they can. They have the tendency to increase the capital (profit) by re-investing the accumulated capital. We argue that Bangladeshi RMG factory owners increased workers’ wages, they also increased working hours, production targets, and reduced breaks between works so that they can accumulate high profit/capital from RMG work (be that exploiting workers’ labour). Thus, Bangladeshi RMG factory owners are accumulating high profits even after the implementation of the new minimum wages by introducing new types of exploitation cycles, e.g. imposing additional working hours, extended production targets, etc. The implementation of the minimum wages can be considered as an eye opener for the international donor community, International Labour Organization (ILO), as well as for the global supply chain, as workers’ real benefits from it remained far from reality.

It is important to remember that it is not only factory owners involved in maximizing profit, rather international brands/buyers are also a big part of this issue. For example, while visiting a factory during the data collection period, the manager of a factory explained how international brands make huge profits from clothing products. He showed a shirt with a tag of the American brand, with the printed price of $69. He described the factory as receiving only $5 for the shirt from the international company. The $64 gap remains in the pocket of that international brand, at the expense of the producers, that is, the workers whose physical and psychological health are compromised. Instead of investing money to ensure a decent life for the workers, the international brands/buyers are more interested in keeping pressure on the factory owners to make the factory compliant, improve their working conditions, not employ child labour, and so on so that they can continue to make big profits from the industry (Ahmed and Nathan 2016; Muhammad 2015; Absar 2001). They often threaten to cancel buying orders if a particular factory fails to maintain those conditions imposed. This begs the questions to be asked, are the international brands willing to pay a bit more to ensure a better workplace conditions or a better life for the workers?

It is time for those who purchase fashion/clothing to fully consider the makers of their fashions/clothes. If every individual customer pays 50 cents extra while buying their clothes, the international labour welfare organizations such as ILO may use that money for the overall betterment of the workers, be that by paying a bit extra money monthly/annually, to ensure a better workplace environment, and to provide them with free and quality healthcare facilities. The recent outbreak of COVID-19 has shown how individual behaviour, choices, and habits could impact the whole RMG global supply chain. Due to the fear of COVID-19 infection, individuals across the globe suddenly ceased usual shopping behaviours and/or shopping centres closed. Thus, “no purchase” from the individual customers forced the international brands/buyers to postpone or cancel the agreed clothing orders, which resulted in factory shutdowns without continuing the wages to RMG workers and they became jobless. That is to say, greater awareness regarding the wage and minimum standard life of the clothing makers should be initiated from the individual customer level which will force the international brands and local RMG factory owners to work for the overall betterment of the RMG workers. Lastly, the future minimum wages for the RMG workers need to comply with the universal basic income (UBI), so that the existing income inequality can be abolished (Sharma 2017). The movement to reform the labour market in the neighbouring countries such as India started a long time ago (Sharma 2006), which has become extremely timely now in relation to the Bangladeshi RMG industry. Implementation of the existing labour rights-related laws, workers’ active participation in trade union activities, and actual benefits of the newly executed wages are required to be ensured/safeguarded by the relevant agencies in Bangladesh.

## Limitations

While this study makes a significant contribution to academia on the impact of the new minimum wages on the RMG workers, a few limitations should be acknowledged. This study was conducted among fifteen in-depth interview participants recruited from Dhaka and Chattogram cities and may not reflect wage-related experiences of the RMG industry across all of Bangladesh. Opinions of different stakeholders (such as factory management, government officials, and the members of BGMEA) regarding the study findings were not considered in this research. Thus, findings may be considered underreported and need to be used carefully. In addition, this study could not explore whether international brands increased the prices of garment products to cover the increase in workers’ wages. However, the team of this research is made of sociologists, social workers, and mental health professionals, who are experts and trained to expose and uncover this multifaceted topic.

## Conclusions

The impacts of the new minimum wage, in relation to RMG workers’ health, well-being, and due rights, focused in this study obviously do not exhaust all the possible factors that shape the utmost scenario of all Bangladeshi RMG factories. By focusing on the basic outcomes of the newly implemented minimum wages and linking them with workers’ expectations, feelings, and reactions, we have gained some insights into how and in what capacity RMG workers of Bangladesh are continuing to work. No doubt that this sector has provided millions of jobs, for workers who had few job opportunities. These workers are contributing enormously to the economic development of the country by bringing major foreign exchange. International clothing brands and the local factory owners must change their high profit-taking mindset and reconsider investing a little more money to provide a safe and healthy working environment as well as minimum standard living rights for the RMG industry workers. They should realize that a new and or upgraded wage is a right for the workers. Workers should not be abused and compelled to increase production targets as they are paid more than the previous gross salary. State, international brands, and ILO must collaboratively monitor workers’ concerns and should take necessary actions against factory owners who fail to ensure a safe and healthy work environment for the workers. This study warns the public that there is a long way to go to ensure decent working conditions in the Bangladesh RMG sector.

Different working groups, national and international policymakers, NGOs/INGOs, labour/trade unions, garment factory owners’ associations, and the individual clothing/fashion wearer may use the findings of this study to take proper steps to reduce the negative impacts of the future implementation of new minimum wages. Workers needed to be articulated with the trade union activities and require necessary training to be aware of their rights and workplace exploitations as non-unionized workers have less bargaining power regarding their wages and rightful facilities at the workplace (Millea et al. 2017). Researchers working on similar topics may consider the findings of this research as the pathway to explore other related problems. In addition, the findings of the study can be used as ground-breaking evidence to propose the need to investigate workers’ wage-related problems in the RMG sector located in the South and Southeast Asian regions [which house the highest number of RMG factories in the globe (Kabir et al. 2019a, b)] because workers of these regions work in the same work setup/settings.

## Data Availability

Data sharing is not applicable to this article.
